# Asymmetric Oxygen Vacancies: the Intrinsic Redox Active Sites in Metal Oxide Catalysts

**DOI:** 10.1002/advs.201901970

**Published:** 2019-12-05

**Authors:** Kai Yu, Lan‐Lan Lou, Shuangxi Liu, Wuzong Zhou

**Affiliations:** ^1^ MOE Key Laboratory of Pollution Processes and Environmental Criteria Tianjin Key Laboratory of Environmental Technology for Complex Trans‐Media Pollution College of Environmental Science and Engineering Nankai University Tianjin 300350 China; ^2^ School of Materials Science and Engineering & National Institute of Advanced Materials Nankai University Tianjin 300350 China; ^3^ School of Chemistry University of St Andrews St Andrews KY16 9ST UK

**Keywords:** asymmetric oxygen vacancies, interfacial catalysts, mixed oxides, redox active sites, single‐atom catalysts

## Abstract

To identify the intrinsic active sites in oxides or oxide supported catalysts is a research frontier in the fields of heterogeneous catalysis and material science. In particular, the role of oxygen vacancies on the redox properties of oxide catalysts is still not fully understood. Herein, some relevant research dealing with M_1_–O–M_2_ or M_1_–□–M_2_ linkages as active sites in mixed oxides, in oxide supported single‐atom catalysts, and at metal/oxide interfaces of oxide supported nanometal catalysts for various reaction systems is reviewed. It is found that the catalytic activity of these oxides not only depends on the amounts of oxygen vacancies and metastable cations but also shows a significant influence from the local environment of the active sites, in particular, the symmetry of the oxygen vacancies. Based on the recent progress in the relevant fields, an “asymmetric oxygen vacancy site” is introduced, which indicates an oxygen vacancy with an asymmetric coordination of cations, making oxygen “easy come, easy go,” i.e., more reactive in redox reactions. The establishment of this new mechanism would shed light on the future investigation of the intrinsic active sites in oxide and oxide supported catalysts.

## Graphical abstract

Asymmetric oxygen vacancies in metal oxide catalysts act as intrinsic catalytic active sites for redox reactions by making oxygen species easy come, easy go. Some relevant work focused on the active sites with asymmetric bonding situations in doped oxides, in oxide stabilized single‐atom catalysts, and at metal/oxide interface is reviewed.

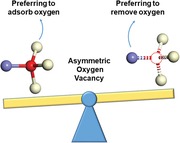

## 1 Introduction

Metal oxide catalysts have been widely investigated in laboratory and in industry because of their excellent activity, selectivity and stability in many important reactions,^1^ especially in some redox reactions, such as CO oxidation,^2^ water–gas shift (WGS) reaction,^3^ selective catalytic reduction of NO*_x_*,^4^ oxidation of volatile organic compounds (VOCs),^5^ and soot combustion.^6^, ^7^ It is commonly known that the catalytic reaction takes place at defect sites of these oxide catalysts.^8^ However, it is not always found a monotonous relation between the catalytic performance and the concentration of a certain kind of defect sites, such as oxygen vacancies,^9^, ^10^ lattice distortion^11^ and defect generated Lewis acid/base sites,^12^, ^13^ as well as metastable valence states of cations.^14^

As discussed by McFarland and Metiu,^15^ heterogeneous catalytic reactions are run under steady‐state conditions instead of at equilibrium. It is difficult to accurately describe the catalytic performance of oxide catalysts using the property parameters of as‐prepared catalysts, which are more likely to be in thermodynamic equilibrium during the preparation process. For example, according to many reports, the redox properties of catalysts were believed to relate to the amount of oxygen vacancies (regarded as active sites) in oxide catalysts. However, the steady‐state concentration of oxygen vacancies is related to the rate of vacancy formation and that of vacancy vanishing. In other words, a real active oxygen vacancy should have high abilities of adsorbing the reactant molecules and desorbing the product molecules, allowing molecules easy come, easy go. To keep a balance of these two opposite abilities, the local environment of the oxygen vacancy plays an important role.

It has been well accepted that doping of low‐valence dopants in oxides can create oxygen vacancies due to a charge compensation effect,^16^, ^17^ accompanied by improved oxygen activation ability, which is usually considered as the key factor in promoting catalytic activity for redox reactions. However, when the oxides are doped by same‐valence or high‐valence dopants, the effect of doping on their catalytic performance will become much more complicated. Fortunately, for most oxidation reactions catalyzed by metal oxides, the lattice oxygen atoms at/near the surface of oxides are the active species based on a generally accepted Mars−van Krevelen (MvK) mechanism.^18^ Therefore, the bonding situations of these surface oxygen atoms should be the key factors affecting the catalytic performance.

In the last few years, researchers have paid more attention to the chemical environment of oxygen vacancies, including site symmetry, bonding strength, etc., rather than the concentration of the vacancies only. Herein, we highlight some recent researches focused on the intrinsic active sites with asymmetric bonding situations in mixed metal oxide catalysts and oxide stabilized single‐atom catalysts (SACs), as well as on the interfacial active sites in between oxide and metal. We will see that the local environment of oxygen vacancies is crucial to the catalytic performance of oxide catalysts. In particular, asymmetric oxygen/vacancy sites can often improve the catalytic performance. We expect this short review may shed light on future identification of intrinsic active sites in oxide and oxide supported catalysts, and microstructural tailoring of active sites for various reactions.

## 2 Asymmetric Oxygen Vacancies in Mixed Oxide Catalysts

### 2.1 What Is the Asymmetric Oxygen Vacancy

**Figure 1:**
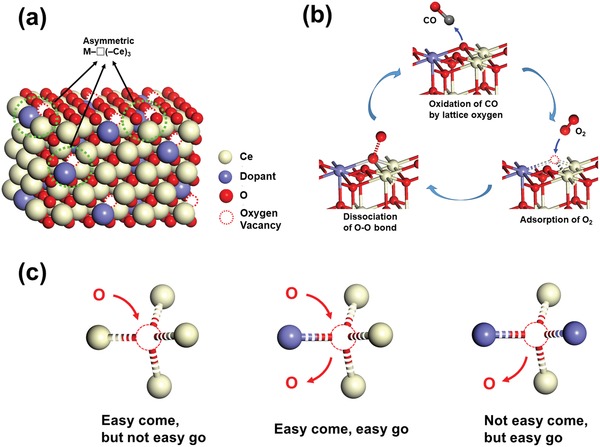
a) The asymmetric oxygen vacancy sites of M–□(–Ce)_3_ in doped ceria. b) The redox catalytic cycle on an asymmetric oxygen vacancy site using aerobic oxidation of CO as an example. c) Schematic diagram of relationship between the microstructure of oxygen vacancy and its redox property.

In a pure oxide, oxygen ions or vacancies usually have a high symmetric coordination with cations. For example, in the fluorite‐type bulk structure of CeO_2_, oxygen anions are tetrahedrally coordinated by four Ce^4+^ cations. A simple method to break down the site symmetry of oxygen/vacancy is to substitute one of the cerium cations, forming an asymmetric site M–O(–Ce)_3_ or M–□(–Ce)_3_, where □ represents an oxygen vacancy (**Figure** **1**a). These sites, coordinated with different cations and expressed as a simplified term of M_1_–O–M_2_ or M_1_–□–M_2_, can be designated as “asymmetric oxygen vacancies,” which can act as active sites in various redox reactions^19^, ^20^, ^21^ involving alternative formation of M_1_–O–M_2_ and M_1_–□–M_2_ (Figure 1b). In these asymmetric sites, there are two conflicting influences from at least two different surrounding cations. One cation can promote the adsorption of oxygen and another favors a vacancy, resulting in two metastable states with and without oxygen occupancy, respectively. The oxygen in these sites is highly active and exhibits promoted activity in several redox reactions. A few examples below demonstrate how asymmetric oxygen vacancies are generated and how they affect the catalytic behaviors of the oxides.

### 2.2 Characterization of Asymmetric Oxygen Vacancies

Although it is challenging to provide direct proof of the existence of asymmetric oxygen vacancies in mixed oxides through electron microscopy, some spectral characterizations can give some indirect evidences. Matsui and co‐workers^22^, ^23^ studied the defect structures in trivalent cation (Gd^3+^ and Y^3+^) doped CeO_2_ materials using extended X‐ray absorption fine structure (EXAFS) and X‐ray absorption near edge structure (XANES) spectroscopy, suggesting that oxygen vacancies introduced by doping were located around both Ce and dopant ions. When the concentration of the dopant is low, guest cations were separated, which was also detected by Moog et al.^24^ in Fe^3+^ doped CeO_2_. With an increase of doping level in CeO_2_, the Ce—O, Y—O and Gd—O bond lengths decreased, suggesting the formation of clusters containing two dopant ions and one oxygen vacancy and/or four dopant ions and two oxygen vacancies.

The local structure of mixed oxides can also be investigated by solid state nuclear magnetic resonance (ssNMR) techniques. Kim and Stebbins^25^ reported the vacancy and cation distribution in Y‐doped CeO_2_ using high‐resolution ^89^Y and ^17^O ssNMR spectroscopy. It was found, based on the ^89^Y spectra, that the average coordination number (CN) of Y was smaller than that of Ce, suggesting a strong association between oxygen vacancies and the dopant cations. The ^17^O NMR characterization of Y‐doped CeO_2_ indicated the existence of oxygen atoms bonding with four Ce cations, three Ce and one Y cations, and two Ce and two Y cations. The similar results are also found in ^17^O ssNMR study of La doped CeO_2_ 26 and Y doped ZrO_2_.^27^ These characterization results indicate the existence of asymmetric oxygen vacancy sites in doped oxides. In particular, doped CeO_2_ catalysts have been extensively investigated in the recent years and a further discussion on more details of these mixed oxides is given below.

### 2.3 Asymmetric Oxygen Vacancy in Doped CeO_2_

CeO_2_ is one of the most popular rare earth oxides and has been widely investigated as catalysts or catalyst carriers for redox reactions.^28^ Many CeO_2_‐based mixed oxides have been reported as efficient catalysts for various reaction systems and their catalytically active sites been discussed. It is of great interest to review the formation of asymmetric oxygen vacancies in CeO_2_ and their role in the catalytic performances.

In the Bi‐doped CeO_2_ solid solution materials, when an octacoordinated Ce^4+^ is replaced by a hexacoordinated Bi^3+^, the asymmetrical oxygen vacancies, Bi–□(–Ce)_3_, will be created neighboring the Bi^3+^, which is favorable to Bi^3+^ as concerning the stabilized coordination number. On the other hand, these oxygen vacancies prefer to be filled by adsorbed oxygen species if a local charge balance and the most stable coordination of Ce^4+^ are considered. Therefore, compared with symmetric Ce–□(–Ce)_3_ site in which the oxygen vacancy is easily oxygenated but difficult to release oxygen, the asymmetric bonding situation of Bi–□(–Ce)_3_ or Bi–O(–Ce)_3_ makes oxygen “easy come, easy go” (Figure 1c), and these asymmetric sites can be identified as the intrinsic active sites in redox reaction.^19^ This conclusion was made based on the observation that the catalytic activity did not increase monotonically with the concentration of Bi and therefore the amount of oxygen vacancies. The best catalytic performance appeared at Ce_0.8_Bi_0.2_O_2−δ_. Further increase of the Bi content to Ce_0.7_Bi_0.3_O_2−δ_ and Ce_0.5_Bi_0.5_O_2−δ_, a significant reduction of catalytic activity was observed,^29^ implying that the catalytic activity not only depends on the amount of oxygen vacancies, but also depends on the local environment of the vacancy sites, since the site symmetry of the oxygen vacancies increased when new clusters containing multiple Bi^3+^ cations formed at high concentration of the dopant.

When the doping level is low (theoretically < 25%), the dopant cations will evenly distribute in the structure, i.e., not forming Bi–O–Bi bonding. All the oxygen vacancies associating with Bi are asymmetric Bi–□(–Ce)_3_. On the particle surface, the coordination number would be reduced, forming Bi–□(–Ce)_2_ and Bi–□–Ce clusters. All these clusters offer the sites that can easily adsorb and desorb oxygen‐containing chemical species. With increasing the Bi‐doping, some Bi‐rich clusters will form according to the “like with like” tendency in solid solutions.^30^, ^31^ All these clusters largely reduced the adsorption capability of the oxygen vacancies (Figure 1c). What we observed at this stage was that the catalytic activity of Bi‐doped CeO_2_ decreased with increasing the Bi doping, in pace with an increase of the concentration of oxygen vacancies.

Obviously, substitution of Ce in CeO_2_ by other trivalent cations would also generate asymmetric oxygen vacancies. For example, Reddy et al.^32^ synthesized nanocrystalline Ce_0.8_La_0.2_O_2−δ_ solid solution and used it as a catalyst for CO oxidation. It was proposed that the oxidation of CO took place via the MvK mechanism, i.e., CO took one lattice oxygen away, leaving a vacancy, which would then be filled by oxygen. Oxygen on the particle surface coordinated by mixed cations, such as La–O(–Ce)_3_, La–O(–Ce)_2_ and La–O–Ce, would be much easier to be removed by CO, when charge balance at La^3+^ and its stable coordination of six oxygen are considered. On the other hand, the oxygen vacancies would be easily filled by oxygen when stabilities of 4+ charge and eight coordination at Ce cations are considered. Unfortunately, Reddy et al. only produced one composition of Ce_0.8_La_0.2_O_2−δ_ and only compared catalytic activity of this solid solution with Zr doped ceria. The latter had much less oxygen vacancies due to the 4+ charge of Zr and showed a lower activity. The authors attributed the improvement of catalytic activity of Ce_0.8_La_0.2_O_2−δ_ to reduced particle size, increased surface area, and increased oxygen vacancies. If a study of catalytic performance as a function of the La content was carried out, the effect of the asymmetric oxygen vacancies would be probably revealed. From these examples, it can be concluded that the relatively stable CN of the cations plays a crucial role in promoting catalytic activity of asymmetric oxygen vacancies. The cations with relatively higher stable CN prefer to adsorb oxygen and the cations with relatively lower stable CN tend to release oxygen from these sites.

Oxygen vacancies in ceria can also be generated by many other low charged cations. Lee et al.^33^ synthesized doped ceria using transition metals, TM*_x_*Ce_1−_ *_x_*O_2−δ_, TM = Mn, Ni, Co or Fe, and studied catalytic properties of the solid solutions using Mn*_x_*Ce_1−_ *_x_*O_2−δ_ as an example. The low‐charged Mn^2+^ can generate relatively stable oxygen vacancies. The number of the vacancies should increase with the doping level of Mn^2+^. The solid solutions exhibited improved catalytic activity for CO oxidation compared with pure ceria. However, such an improvement of catalytic properties did not change monotonically with the doping level. As detected by the authors, the catalytic activity of CO oxidation increased from pure ceria to Ce_0.92_Mn_0.08_O_2−δ_, but reduced with further increase of the Mn^2+^ doping, e.g., Ce_0.82_Mn_0.18_O_2−δ_. The phenomenon is similar to the Bi‐doped ceria catalysts for aerobic oxidation reactions.^19^ At a low level of doping, Mn^2+^ are well separated and form asymmetric oxygen vacancies, e.g., Mn–O(–Ce)_3_. Because the difference between Mn^2+^ and Ce^4+^ cations is much more significant than that between Bi^3+^ and Ce^4+^, in the solid solution of Mn*_x_*Ce_1−_ *_x_*O_2−δ_, oxygen vacancies with multiple Mn coordination would form more easily.

It is noticed that determination of the local structures around the dopant cations in the Mn‐doped ceria is often difficult, since the oxidation state of Mn varies from 4+ to 3+, and to 2+, especially when the concentration of Mn is high. Unlike Bi^3+^ with a constant oxidation state, Mn often exists in a mixed oxidation state in ceria, which can reduce the symmetry of the vacancy sites at different levels. Ramana et al.^34^ found that Ce_0.7_Mn_0.3_O_2−δ_ solid solution shows superior vanillyl alcohol oxidation activity in comparison with Ce_0.8_Mn_0.2_O_2−δ_. With an even higher Mn doping, Ce_0.5_Mn_0.5_O_2‐δ_ was found to have a very high catalytic activity for CO oxidation.^35^ It also exhibited the highest catalytic activity for the selective oxidation of hydrocarbons. The authors suggested that the formation of Ce–O–Mn bond would reduce the Coulomb interaction of Mn^δ+^–O^γ−^ or Ce^δ+^–O^γ−^, making oxygen more reactive. When the symmetry of the oxygen sites is considered, the relatively higher asymmetry of the sites would indeed allow oxygen to be removed easily, but more difficult to be adsorbed. We believe this is why high O_2_ pressure was applied to re‐fill the vacancies in that work.^35^ From these reports, we can find that the oxidation state of cations associated with asymmetric oxygen vacancies is an important factor to influence the activity of these sites. The local charge imbalance can promote the reactivity of oxygen in these asymmetric active sites, and the various oxidation states of cations can contribute to the localization of the electrons in the asymmetric oxygen active sites and facilitate the catalytic cycle in these sites, which has been discussed by Murgida et al. through density functional theory (DFT) calculation.^36^

In addition, the electronic structure of cations in asymmetric oxygen vacancy sites is also regarded as a key factor to determine the reactivity of the oxygen in these sites. A recent DFT calculation for SrMO_3_ perovskites^37^ shows a clear correlation between *d*‐electron count of M cations and the vacancy formation energy, i.e., the reactivity of lattice oxygen. The M cations with larger *d*‐electron count exhibit higher reactivity of lattice oxygen in M—O bonds. Moreover, the energy band structure of oxides determines the extent of electron delocalization from the oxygen vacancies,^38^ which is beneficial for the stabilization of oxygen vacancies.

Doping ceria with noble metal cations, such as Au, Ag, Pt, Pd, and Ru ions, can more efficiently increase the reactivity of oxygen near the dopant cations. Fu et al.^3^ prepared a series of ceria‐based Au or Pt catalysts for WGS reaction (CO + H_2_O ↔ CO_2_ + H_2_). It was found that nonmetallic Au or Pt species strongly associated with surface Ce—O groups, i.e., forming Au–O–Ce or Pt–O–Ce linkage, are responsible for the activity by creating additional oxygen vacancies on the surface of ceria, while metallic Au or Pt nanoparticles do not participate in the reaction. The principal oxidation states of the guest cations are Au^+^ and Pt^2+^. More importantly, the authors pointed out that the oxygen vacancies would be generated if Au or Pt occupied Ce sites in ceria. It is not surprising because the oxygen vacancies associated with Au or Pt cations are asymmetric and the oxygen species occupying these sites will be significantly activated.

Shapovalov and Metiu performed DFT calculations for the CeO_2_ (111) surface doped with Au, Ag, or Cu, and found that oxygen associated with the dopant can be activated.^39^ Notably lower formation energies of oxygen vacancy (*E*_ov,_ e.g., −0.36 eV for Au doped CeO_2_) in comparison with undoped CeO_2_ (+3.01 eV) can be achieved. It is interesting to read the authors' argument: to be a good catalyst for the CO oxidation, a doped oxide must achieve a balance between two conflicting requirements, that is the adsorption and desorption of oxygen. It must make surface oxygen reactive but not so much that it will hinder the healing of the oxygen vacancies created by the oxidation reaction.^39^ Asymmetric oxygen sites coordinated with mixed metal cations, e.g., Au–O(–Ce)_2_ on the CeO_2_(111) can offer such active centers. Pt doping into CeO_2_ can also activate its neighboring surface oxygen atoms, making them more chemically reactive toward methane oxidation. Moreover, the catalyst is more active as it contains more ionic Pt.^40^ These examples support the proposed effect of asymmetric oxygen vacancies on catalytic performance, as doping with these noble metal cations would enhance greater distortion of the sites in question, in comparison with trivalent cations such as Bi^3+^ and La^3+^, etc.

Besides the experimental investigation, the catalytic cycle process of asymmetric oxygen vacancy active sites in redox reactions was also studied by computational methods. Camellone and Fabris^21^ proposed, based on the DFT+U calculations, a three‐step catalytic cycle on Ce_1−_ *_x_*Au*_x_*O_2−δ_ solid solutions for CO oxidation, which occurred on the oxygen vacancy sites next to Au^3+^ and Ce^3+^ (Figure 1b). (I) A lattice oxygen next to Au^3+^ and Ce^4+^, e.g., in asymmetric sites of Au^3+^–O–Ce^4+^, participates in the oxidation of CO to CO_2_, leading to the formation of an oxygen vacancy and the reduction of Ce^4+^, e.g., Au^3+^–□–Ce^3+^. (II) The adsorption of O_2_ molecule at this oxygen vacancy site, leading to the reoxidation of Ce^3+^ to Ce^4+^. (III) The adsorbed O_2_ molecule dissociates and leaves one oxygen atom at the active site. Finally, the step (I) is repeated, making a cycle. The similar three‐step catalytic cycle was also proposed by Chen for CO oxidation on Ru‐doped CeO_2_ (111) surface by means of first‐principles calculations.^41^

### 2.4 Asymmetric Oxygen Vacancy in Other Doped Oxides

Besides the ceria based solid solutions, the asymmetrical oxygen vacancy sites also exist in many other solid solution oxide catalysts. For example, Cu^2+^ doped Co_3_O_4_ nanowires exhibited notably improved catalytic activity for CO oxidation compared with pure Co_3_O_4_.^42^ DFT calculations showed that the oxygen vacancy was more easily generated in the bonding of Co^3+^–O–Cu^2+^ compared with Co^3+^–O–Co^2+^, implying the higher reactivity of oxygen in the former.

The asymmetric bonding enhanced active lattice oxygen ions were also observed in divalent metal cation M^2+^‐doped MnO*_x_* materials (M^2+^ = Ni^2+^, Co^2+^, or Fe^2+^)^43^ for aerobic oxidation of 5‐hydroxymethylfurfural. Compared with symmetric Mn^4+^–O(–Mn^4+^)_2_ clusters, the oxygen atoms in asymmetric M^3+^–O(–Mn^4+^)_2_ clusters are more favorable to participate in the oxidation reaction by reducing the active sites to M^2+^–□(–Mn^3+^)_2_, in which the oxygen vacancies can be easily filled by O_2_ adsorption and dissociation. Consequently, active oxygen in these asymmetric active sites can be “easy come, easy go.”

Dong and co‐workers reported a series of CuO‐based mixed oxide catalysts for NO reduction by CO and the M_1_–O–M_2_ and M_1_–□–M_2_ like active sites were proposed,^44^, ^45^, ^46^, ^47^ offering excellent examples to demonstrate enhancement of catalytic activity by the formation of asymmetric oxygen vacancies through the doping of high oxidation state cations. Cu and Mn in the CuO‐Mn_2_O_3_/γ‐Al_2_O_3_ system were evenly distributed at the γ‐Al_2_O_3_ surface and occupied the octahedral vacant sites of Al.^44^ When CuO and Mn_2_O_3_ were not mixed, symmetric clusters Cu–O–Cu in CuO/γ‐Al_2_O_3_ and Mn–O–Mn in Mn_2_O_3_/γ‐Al_2_O_3_ showed very low activities in reduction of NO with CO. When mixed CuO and Mn_2_O_3_ were loaded on γ‐Al_2_O_3_, the formation of asymmetric oxygen sites coordinated by both Cu and Mn, denoted as Cu–O–Mn, greatly improved the catalytic activity. The CuO‐MnO*_x_*/TiO_2_ showed a similar behavior.^45^ In these oxides, Mn prefers a six‐coordination with oxygen, but Cu intends to have a four‐coordination. Consequently, Cu takes the main responsibility of introducing oxygen vacancies, and therefore activating the oxygen atoms in the Cu–O–Mn sites. On the other hand, Mn makes the adsorption of oxygen species easier. Similar results were also found in supported CuO–VO*_x_* 46 and CuO–CoO^47^ mixed oxide catalysts.

## 3 Asymmetric Oxygen Vacancies in Single‐Atom Catalysts

In mixed oxides, the asymmetric oxygen vacancy sites disperse throughout the oxides. The sites located in the interior of the oxides would improve the mobility of the lattice oxygen and benefit their redox property. However, only those located in the surface region can directly participate in catalytic reactions. Therefore, constructing asymmetric active sites at oxide surface should be a more atom‐economic strategy to fabricate highly efficient catalysts.

**Figure 2:**
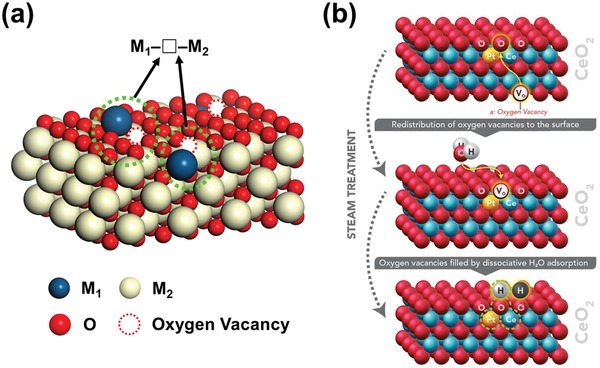
a) The asymmetric oxygen vacancy sites on the surface of SACs. b) The active site of Pt–O–Ce in Pt/CeO_2_ created by the steam treatment. Reproduced with permission.^49^ Copyright 2017, American Association for the Advancement of Science.

SACs have recently received much attention and become a new frontier in the heterogeneous catalysis field,^48^ in which the atomically dispersed metals stabilized on the surface of a substrate often act as the isolated catalytic active sites. In some cases, however, the reaction may take place at the surrounding atoms, including oxygen and the metal cations in the substrate, rather than on the single‐atomic metal sites. It is of great interest to see if we can find active asymmetric oxygen vacancy sites in these materials (**Figure** **2**a). The answer is positive. Nie et al.^49^ reported that the steam treatment of atomically dispersed Pt^2+^/CeO_2_ catalysts can effectively create active surface lattice oxygen species for low‐temperature CO oxidation (Figure 2b). DFT calculations showed that the activation barrier of CO on steam treated catalyst is nearly half to that of untreated catalyst. This new type of active lattice oxygen, located in the vicinity of Pt^2+^, was generated by filling the surface oxygen vacancies (Pt–□–Ce) that are derived from the substitution of Pt^2+^ for Ce^4+^ by the dissociative H_2_O adsorption. The principle of enhancement of catalytic activity is similar to that in doped CeO_2_ as discussed above. The relatively low oxidation state and low favorable coordination number of Pt stabilize the vacancies in the asymmetric sites of Pt–□–Ce or activate oxygen atoms in Pt–O–Ce. The beauty of this material is that almost all Pt atoms are at oxide surface and each Pt can activate two or more surrounding oxygen atoms.

Similarly, Li et al.^50^ reported that the single‐atom Au_1_/CeO_2_ catalyst exhibited notably increased turnover frequency values for aerobic oxidation of benzyl alcohol compared with CeO_2_ supported Au nanocatalyst, which is mainly attributed to much more activated lattice oxygen atoms generated by highly dispersed Au single‐atoms. In addition, this catalyst was of excellent reusability and no aggregation of Au atoms occurred in the recycling test.

## 4 Asymmetric Oxygen Vacancies at Metal/Oxide Interface

**Figure 3:**
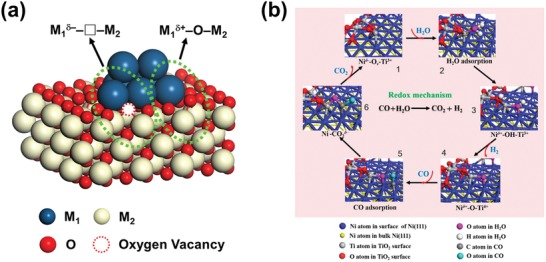
a) The asymmetric oxygen vacancy sites at edge of the interface between oxide and metal. b) Schematic diagram of WGS reaction over Ni@TiO_2−_ *_x_* catalyst based on a redox mechanism. Reproduced with permission.^57^ Copyright 2018, American Chemical Society.

Recently, the interfacial active sites on oxide‐supported metal catalysts or oxide‐on‐metal catalysts are considered as a hot topic in heterogeneous catalysis field.^51^ The oxygen atoms at metal/oxide interface with asymmetric bonding situation and connected with coordinatively unsaturated cations are highly active for catalytic oxidation reactions.^52^, ^53^ In some recent reports, the interfacial oxygen species/vacancies with an asymmetric bonding situation between oxide and metal (**Figure** **3**a) were regarded as the intrinsic active sites for CO oxidation and WGS reaction.

Bao and co‐workers^54^ applied Pt(111) supported FeO nanoislands, [FeO_1−_ *_x_*/Pt(111)] as catalysts for CO oxidation and found that the interface‐confined coordinatively unsaturated ferrous sites and neighboring Pt atoms, that is, Fe^2+^–O–Pt^δ+^ sites, are the active centers for O_2_ activation with low adsorption energy for O_2_ molecules and low reaction barrier for O_2_ dissociation to atomic O. From this work, we see that active asymmetric oxygen vacancy sites can be achieved not only in surface layers of a doped metal oxide or in SACs, but also in metal/oxide interface, forming a M_1_–O–M_2_ or M_1_–□–M_2_ bridge‐like structure. The oxygen atoms at these sites are much more active than the lattice oxygen in metal oxide, in particular, the oxygen sites with lower CN at the edges of metal/oxide interface. But the oxygen sites underneath the supported nanocluster might be deactivated even though they are asymmetric.^55^ It can be explained by the increased CN of oxygen atoms, which has been proved to be directly correlated with the reactivity of lattice oxygens.^56^

Xu et al.^57^ reported the interfacial oxygen vacancy active sites, Ni^δ−^–□–Ti^3+^, on Ni@TiO_2−_ *_x_* catalyst for WGS reaction. The redox mechanism on these active sites was proposed (Figure 3b). It was observed that the catalytic cycle between Ni^δ−^–□–Ti^3+^ and Ni^δ+^–O–Ti^4+^ plays the key role in the oxidation of CO and dissociation of H_2_O. DFT calculations showed that the activation barrier of H_2_O dissociation on the interfacial asymmetric Ni^δ−^–□–Ti^3+^ sites (≈0.35 eV) was notably lower than those on the metallic Ni(111) surface (≈0.87 eV) and on the symmetric Ti–□–Ti sites on TiO_2−_ *_x_*(101) surface (≈0.42 eV).

**Figure 4:**
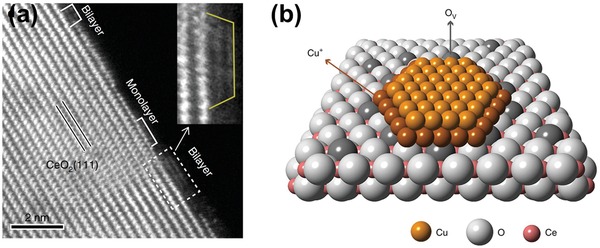
Atomic structure of the Cu/CeO_2_ interfacial perimeter. a) The atom‐resolved HAADF‐STEM images of copper clusters on ceria rods. b) A schematic illustration of the typical Cu bilayer on ceria. Reproduced with permission.^58^ Copyright 2019, Springer Nature.

Very recently, Chen et al.^58^ synthesized rod‐shaped ceria supported ultrafine copper clusters that presented mainly as bilayers. The atomic structure of the catalytically active sites located at Cu/CeO_2_ interfacial perimeter, in a form of Cu^+^–□–Ce^3+^, for the low‐temperature WGS reaction were directly identified based on a combined microscopic and spectroscopic studies (**Figure** **4**). These active sites showed a synergistic effect during the reaction with the Cu^+^ sites adsorbing CO and the □–Ce^3+^ sites dissociatively activating H_2_O. Analysis of in situ infrared spectra of the catalyst suggested the redox circle between Cu^+^–□–Ce^3+^ and Cu^2+^–O–Ce^4+^ sites upon alternating reductive and oxidative atmospheres.

In the above discussed FeO_1−_ *_x_*/Pt(111), Ni@TiO_2−_ *_x_* and Cu/CeO_2_ catalysts, the active oxygen sites are at the interface between the metal and oxide, and at the edge of supported nanoparticles. These sites normally have only two coordination with a metal atom on one side and a cation in the oxide on the other. Since the oxygen is not at a center of a regular polyhedral coordination, site distortion is not relevant and the space of the sites is quite flexible. The effect of asymmetry of the sites is mainly from the different properties of the two coordinated atoms. For example, in Ni^δ−^–□–Ti^3+^ and Ni^δ+^–O–Ti^4+^ bridges mentioned above, the change of oxidation state of ions is commonly acceptable. The local charge at Ni is the key factor to influence the activity of the sites, but is difficult to be detected.

## 5 Conclusion and Outlook

Oxygen vacancies in metal oxide catalysts are often considered as intrinsic active centers for many important redox reactions. People normally believe that the catalytic activity on these vacancies is a function of the concentrations of the vacancies and the surrounding reduced cations. Recent researches indicate that the activity of the oxygen vacancies also depends on the local environment of the sites. Based on the relevant advances in this field, the concept of “asymmetric oxygen vacancy site” has been proposed, which has two conflicting influences from at least two different surrounding cations. One cation can promote the adsorption of oxygen and another favors a vacancy. These different functions can be made from different sizes, electronic structure, oxidation states, relatively stable coordination numbers of the cations. The strength of these influences can be adjusted to suit different chemical reactions, in principle, to make a balance of adsorption and desorption of oxygen species.

It is interesting to find that asymmetric oxygen vacancies exist not only in mixed oxides, but also in oxide supported SACs, and even at metal/oxide interfaces. More experiments will have to be performed before the mechanisms of the improvement of catalytic activity with asymmetric oxygen vacancies can be fully understood. Future work will also focus on the influence of asymmetric oxygen vacancies on charge transfer in catalysts, in particular, in between metal nanoparticles and mixed oxide substrates. More accurate computational calculation and the characterization at atomic level of the microstructures of the asymmetric oxygen vacancies are also challenging targets.

## Conflict of Interest

The authors declare no conflict of interest.
